# Mutation at *Grassy tiller 1* increases rice yield production and resistance to sheath blight

**DOI:** 10.1007/s44297-024-00025-0

**Published:** 2024-04-03

**Authors:** Shuo Yang, Vikranth Kumar, Xin Tong Jia, Ai ping Zheng, Yuan Hu Xuan

**Affiliations:** 1 College of Plant Protection, Shenyang Agricultural University, Shenyang, 110866 China; 2 Division of Plant Sciences, University of Missouri, Columbia, MO 65211 USA; 3 Department of Plant Protection, Rice Research Institute, Sichuan Agricultural University, Chengdu, China; 4 State Key Laboratory of Elemento-Organic Chemistry and Department of Plant Protection, National Pesticide Engineering Research Center (Tianjin), Nankai University, Tianjin, 300071 China

**Keywords:** GT1, Sheath blight, Resistance, Yield production, Rice

Disease occurrence severely affects crop yield production; however, the signaling pathway controlling yield and immunity are antagonistic that made plant breeding even difficult [1]. Plant trait of tiller, e.g., tiller number, is the key architectural factor that is directly associated with yield production [2]. The previous transcriptome study using sheath blight (ShB) susceptible cultivar (Lemont) and resistant cultivar (Teqing) identified that 2048 genes including *Grassy tiller 1* (*GT1*) differentially expressed upon inoculation of *Rhizoctonia solani* AG1-IA (Fig. 1a) [3]. RT-qPCR analysis confirmed that *GT1* expression level was drastically lower in Teqing than in Lemont (Fig. 1b). Our previous study also showed that *GT1* expression is significantly induced by *R*. *solani* AG1-IA [4]. qRT-PCR results verified that *R. solani*-inoculation significantly induced *GT1* expression after 12, 36 and 48 h, and the highest induction was detected after 36 h of inoculation (Fig. 1c). These results demonstrated that disease-related *GT1* was significantly activated by *R. solani*. Transcriptome analysis using *GT1* RNAi plants determined that *sugar will eventually be exported transporter* (*SWEET*) *2a* and *SWEET3a* were regulated by changes with GT1 expression levels [5]. SWEETs play the major roles in the interaction between host plant and microbe [6, 7]. Transactivation assay and qRT-PCR results confirmed the *OsSWEET2a* (qOsSWEET2a-F: GTGTTCGCTCTAATTGTG; qOsSWEET2a-R: AGAACTCCATATGCGAAG) and *OsSWEET3a* (qOsSWEET3a-F: TTGGTAGTGTTGGTCTAG; qOsSWEET3a-R: AAAGGATCCCTTCCTATG) were visibly activated by GT1(Fig. 1d and e). The DNA fragments immunoprecipitated by anti-GFP antibody from *35S:GFP* and *35S:GT1:GFP* calli together with further ChIP-PCR revealed that GT1 bound to P1 which harboring GT1-binding motif, but not P2 in the *SWEET2a* promoter. Also, GT1 bound to P3, which harboring GT1-binding motif, but not P4 in the *3a* promoter (Fig. 1f and g). Cotransformation of *35S:GT1* with the constructs of *SWEET2a* and *SWEET3a* promoters drive the open reading frame (ORF) sequences of *beta-glucuronidase* (*GUS*), *pSWEET2a-GUS*, *pSWEET3a-GUS*, or GT1-binding motif mutated *mpSWEET2a-GUS* and *mpSWEET3a-GUS* in the protoplast cells identified that GT1 activates *pSWEET2a-GUS* and *pSWEET3a-GUS*, but not *mpSWEET2a-GUS* and *mpSWEET3a-GUS*. (Fig. 1h and i). The results of *R. solani* inoculation demonstrated that *GT1 RNAi* were less susceptible to ShB compared to wild-type control Dongjin (DJ), while *GT1 RNAi* (DJ background) and *gt1*, *sweet2a* and *sweet3a* mutants (Zhonghua 11, ZH11 background) were less susceptible to ShB compared to their corresponding WT control [8] (Fig. 1j- m). These aforesaid results indicated that GT1 regulates rice susceptibility to ShB by activating two gene of *SWEET2a* and *3a* which was consistent with previous research findings [8].

**Fig. 1:**
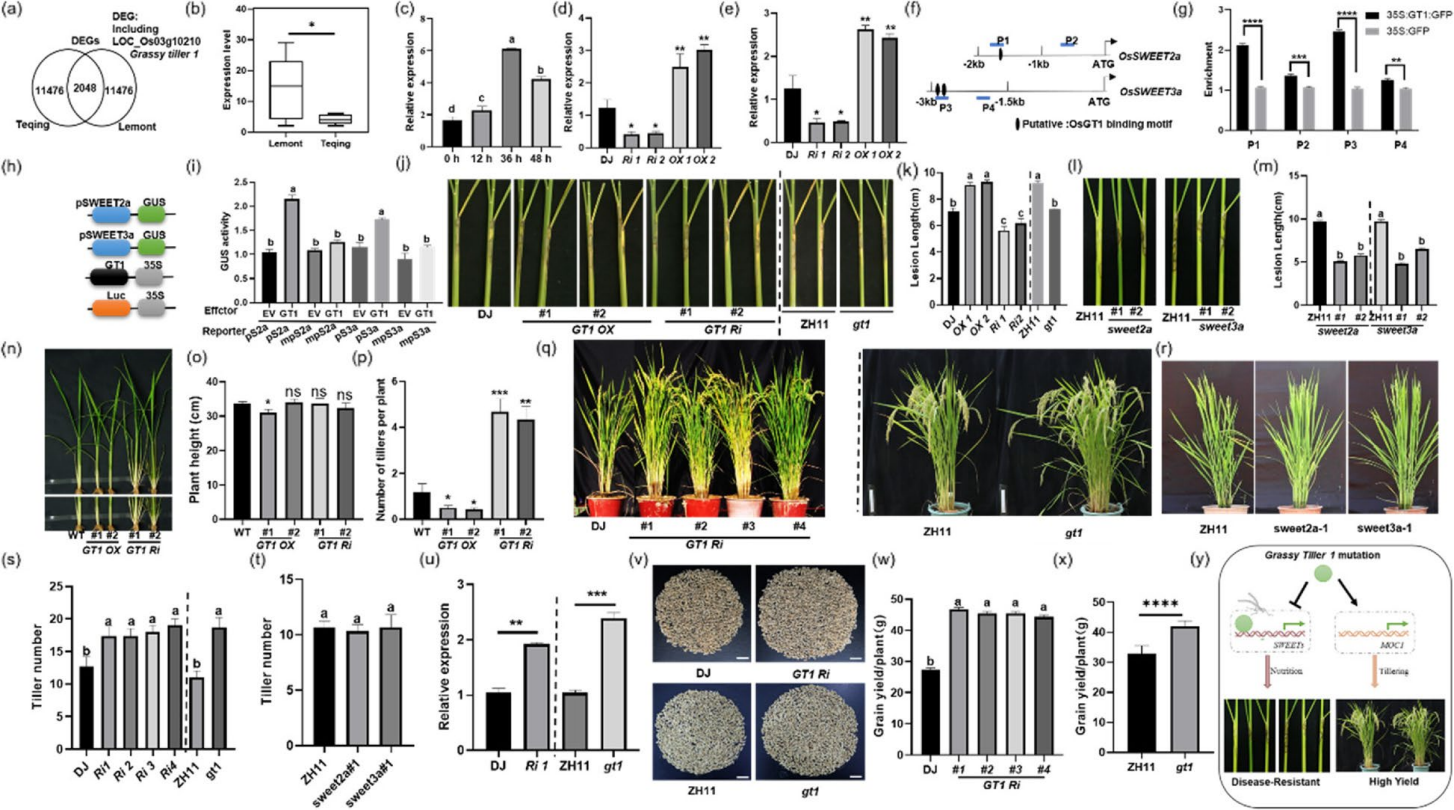
Mutation at *GT1* promotes rice tillering and resistance to ShB. **a** The venn diagrams showing distribution of number of DEGs including *grassy tiller 1* in transcriptome assays of Teqing (resistant variety) and Lemont (susceptible variety) inoculated with *R. solani* at different time points. **b** *GT1* expression levels in susceptible (Lemont) and resistant (Teqing) genotypes were analyzed by RT-qPCR. The statistical significance of differences is represented by an asterisk (student *t*-test). **c** qRT-PCR analyses of *GT1* expression level 0, 12, 36, and 48 h following the inoculation of *R. solani*. **d** Gene expression levels of *SWEET2a* in wild-type control Dongjin (DJ) and two lines of *GT1* RNA interfering lines (*GT1 RNAi #1*, *#2*), and *GT1* overexpressors (*GT1 OX #1*, *#2*) plants. **e** Gene expression levels of *SWEET3a* in DJ, *GT1 RNAi* (*#1*, *#2*), and *GT1 OX* (*#1*, *#2*) plants. **f** Schematic diagram displaying the location of GT1 binding motifs and the probes (P1-P4) in the *SWEET2a* and *SWEET3a* promoters used for the chromatin immunoprecipitation (ChIP) analysis. The black ellipses represent the putative GT1 binding motifs. The blue lines and the letters ‘P’ indicate the locations of the probes. **g** The relative ratio of immunoprecipitated DNA to input DNA was measured through qPCR in *35S:GT1:GFP* and *35S:GFP* transgenic plants. Input DNA was used for data normalization. **h** Schematic diagram showing *pSWEET2a* and *pSWEET3a* or the GT1 binding motif mutated promoter *mpSWEET2a* and *mpSWEET3a* were fused to the *GUS* coding sequences to generate the reporter construct. These reporter constructs were cotransformed with the effector (*35S:GT1*) and GUS activity was analyzed. **i** Transient assay was performed to verify GT1 activation on normal and GT1-binding motif mutated *SWEET2a* (*pS2a* and *mpS2a*) and *SWEET3a* (*pS3a* and *mpS3a*) promoters. *35S:Luc* (luciferase) was used as the internal control to normalize transformation efficiency. **j** The sheaths from the DJ, *GT1 RNAi* (*#1*, *#2*), *GT1 OX* (*#1*, *#2*), ZH11, and *gt1* plants were inoculated with *R. solani*. **k** The lesion lengths on the surface of DJ, *GT1 RNAi* (*#1*, *#2*), *GT1 OX* (*#1*, *#2*), ZH11, and *gt1* plants were measured. **l** The sheaths from the ZH11, *sweet2a* (*#1*, *#2*), and *sweet3a* (*#1*, *#2*) plants were inoculated with *R. solani*. **m** The lesion lengths on the surface of ZH11, *sweet2a* (*#1*, *#2*), and *sweet3a* (*#1*, *#2*) plants were measured. **n** The phenotype of 4-week-old DJ, *GT1 OX* (*#1*, *#2*), and *GT1 RNAi* (*#1*, *#2*) plants. **o** The plant height of 4-week-old DJ, *GT1 OX* (*#1*, *#2*), and *GT1 RNAi* (*#1*, *#2*) plants were measured. **p** The plant height of 4-week-old DJ, *GT1 RNAi* (*#1*, *#2*), and *GT1 OX* (*#1*, *#2*) plants were measured. **q** The number of tillers per plant of 3-month-old DJ, *GT1 RNAi* (*#1*, *#2*, *#3*, and *#4*) plants, ZH11, and *gt1* were calculated. **r** The phenotype of 3-month-old ZH11, *sweet2a-1,* and *sweet3a-1* plants. **s** The number of tillers per plant of 3.5-month-old ZH11, DJ, *gt1*, and *GT1 RNAi* (*#1*, *#2*, *#3,* and *#4*) plants were measured. **t** The number of tillers per plant of 3-month-old ZH11, *sweet2a* (*#1*)*,* and *sweet3a* (*#1*) plants were measured. **u** Expression level of *MOC1* was measured in ZH11, DJ, *gt1*, *GT1 RNAi* (*#1*) plants. **v** The phenotype of filling grain in individual DJ, *GT1 RNAi* (*#1*, *#2*, *#3,* and *#4*) plants and ZH11, *gt1* plants. **w** The grain weight per line was measured in individual DJ and *GT1 RNAi* (*#1*, *#2*, *#3,* and *#4*) plants. **x** The grain weight per plant were measured in individual ZH11, *gt1* plants. **y** Diagram of mutant *GT1* avoiding trade-offs between immunity and yield. *GT1* mutation inhibited the activation of *SWEET2a* and *SWEET3a*, avoiding plant sugar nutrient leakage, and improving disease resistance. In parallel, the increase in *MOC1* expression promotes tillering, a key trait in yield formation, increasing the number of spikes per plant. Ultimately, this strategy avoids the trade-off between yield and immunity via the SWEETs-GT1-MOC1 pathway. Error bars represent the standard deviation with biological repetitions. Different letters or asterisk above the bars designate significant differences (*P* < *0.05*)

GT1 is an ortholog of maize *Grassy tiller 1*, which negatively regulates tillering in DJ [5]. The height difference between *GT1RNAi* and *GT1OX* plants during the seedling stage was not significant compared to WT plants (Fig. 1n-o). Compared to WT, *GT1 OX* plants developed fewer tillers, while *GT1 RNAi* plants developed more tillers (Fig. 1n and p). Also, *gt1* mutant in ZH11 background developed more tillers than ZH11, which is similar with *GT1 RNAi* (*#1*, *#2*, *#3*, and *#4*) tiller phenotype in the DJ background at the mature stage, suggesting that *GT1* might negatively control tillering independent of the cultivar background (Fig. 1q and s). Since GT1 directly activates *SWEET2a* and *SWEET3a*, the possibility of *SWEET2a* and 3a regulation of tillering was examined. However, *sweet2a* (*#1*) and *sweet3a* (*#1*) mutants developed similar number of tillers compared to ZH11 (Fig. 1r and t). Next, we tested the expression level *MONOCULM 1* (*MOC1*), a master regulator of rice tillering [9]. MOC1 expression levels was higher in *gt1* or *GT1 RNAi* plants than in their corresponding WT control (Fig. 1u). To identify the impact of *GT1* on rice yield, the individual plant yield of *GT1 RNAi,* DJ*, gt1*, and ZH11 was measured. The results demonstrated that *GT1 RNAi* and *gt1* plants developed more grains per plant than WT (Fig. 1v-x). As one of the traits of yield formation, tiller outgrowth requires the coordination of elevated CO_2_ conditions and N application rate by modulating multiple glutamate receptors, ammonium transporters, nitrate transporters, and peptide transporters expressions in leaves and SAM tissues [10]. N uptake promotes chlorophyll biosynthesis to improve photosynthesis and sugar production in plants [11]. Previous study has shown that *R. solani* inoculation induced expression of ammonium transporter AMT1, which promotes ammonium uptake and subsequent nitrogen assimilation, e.g., chlorophyll biosynthesis, to improve rice resistance to ShB [12]. In addition, *amt1* mutant was produced lower chlorophyll and more susceptible to ShB as well as developed less tillers compared to wild-type, implying that nutrient transport and photosynthesis might be tightly associated with tillering and defense. *GT1* mutants are also developed more tiller and enhanced rice resistance to ShB; therefore, it might be valuable to examine the GT1 regulation on nutrient uptake and photosynthesis. Taken together, our analyses proved that mutation at a single *GT1* simultaneously increases rice resistance to ShB and tillering. The molecular mechanism suggested that GT1 might activates *SWEET2a* and *3a* to control intracellular sugar contents to promote *R. solani* growth, in parallel, GT1 negatively activates *MOC1* to regulate rice tillering, a key factor in yield formation (Fig. 1y). This finding extended ShB resistant mechanism and provided useful molecular target for resistant breeding avoid trade-off between yield and immunity pathways [13].

## Data Availability

Not applicable.

## References

[1] Ning Y, Liu W, Wang GL. Balancing immunity and yield in crop plants. Trends Plant Sci. 2017;22(12):1069–1079. 29037452 10.1016/j.tplants.2017.09.010

[2] Wang Y, Li J. Rice, rising. Nat Genet. 2008;40:1273–1275. 18957983 10.1038/ng1108-1273

[3] Wang A, Jiang Y, Yamamoto N, Li S, Liang Y, Zou T, Shu X, Jing X, Jiao C, Chen L, Zhang J, Ma L, Deng Q, Wang S, Zhu J, Liu H, Wang L, Huang Y, Li P, Zheng A. Identification of rice (Oryza sativa L.) genes involved in sheath blight resistance via a genome-wide association study. Plant Biotechnol J. 2021;19(8):1553–1566. 33600077 10.1111/pbi.13569 PMC8384605

[4] Yuan D, Xu X, Hong WJ, Wang S, Jia X, Liu Y, Li S, Li Z, Sun Q, Mei Q, Li S, Jung KH, Wei S, Xuan Y. Transcriptome analysis of rice leaves in response to *Rhizoctonia solani* infection and reveals a novel regulatory mechanism. Plant Biotechnol Rep. 2020;14:559–573.

[5] Kumar V, Kim S, Adnan M, Heo J, Jeong J, Priatama R, Lee J, Kim C, Je B, Park S, Xuan Y, Han C. Tiller outgrowth in rice (Oryza sativa L.) is controlled by OsGT1, which acts downstream of FC1 in a PhyB-Independent manner. J Plant Biol. 2021;64:417–430.

[6] Chen LQ, Hou BH, Lalonde S, Takanaga H, Hartung M, Qu X, Guo WJ, Kim JG, Underwood W, Chaudhuri B, Chermak D, Antony G, White F, Somerville S, Mudgett M, Frommer W. Sugar transporters for intercellular exchange and nutrition of pathogens. Nature. 2010;468(7323):527–532. 21107422 10.1038/nature09606 PMC3000469

[7] Bezrutczyk M, Yang J, Eom JS, Prior M, Sosso D, Hartwig T, Szurek B, Oliva R, Vera-Cruz C, White F, Yang B, Frommer W. Sugar flux and signaling in plant-microbe interactions. Plant J. 2018;93(4):675–685. 29160592 10.1111/tpj.13775

[8] Yang S, Fu Y, Zhang Y, Yuan D, Li S, Kumar V, Mei Q, Xuan Y. *Rhizoctonia solani* transcriptional activator interacts with rice WRKY53 and grassy tiller 1 to activate SWEET transporters for nutrition. J Adv Res. 2022;S2090–1232(2):00216–00218. 10.1016/j.jare.2022.10.001 PMC10403663 36252923

[9] Li X, Qian Q, Fu Z, Wang Y, Xiong G, Zeng D, Wang X, Liu X, Teng S, Hiroshi F, Yuan M, Luo D, Han B, Li J. Control of tillering in rice. Nature. 2003;422:618–621. 12687001 10.1038/nature01518

[10] Zhou J, Gao Y, Wang J, Liu C, Wang Z, Lv M, Zhang X, Zhou Y, Dong G, Wang Y, Huang J, Hui D, Yang Z, Yao Y. Elevated atmospheric CO2 concentration triggers redistribution of nitrogen to promote tillering in rice. Plant Environ Interact. 2021;2(3):125–136. 37283862 10.1002/pei3.10046 PMC10168068

[11] Bassi D, Menossi M, Mattiello L. Nitrogen supply influences photosynthesis establishment along the sugarcane leaf. Sci Rep. 2018;8:2327. 29396510 10.1038/s41598-018-20653-1 PMC5797232

[12] Wu X, Yuan D, Chen H, Kumar V, Kang S, Jia B, Xuan Y. Ammonium transporter 1 increases rice resistance to sheath blight by promoting nitrogen assimilation and ethylene signalling. Plant Biotechnol J. 2022;20(6):1085–1097. 35170194 10.1111/pbi.13789 PMC9129087

[13] He Z, Webster S, He Y. Growth-defense trade-offs in plants. Curr Biol. 2022;32(12):R634–R639. 35728544 10.1016/j.cub.2022.04.070

